# Supplementary material for: Onset of Senescence and Steatosis in Hepatocytes as a Consequence of a Shift in the Diacylglycerol/Ceramide Balance at the Plasma Membrane
Source: Cells. 2021 May 21;10(6):1278. doi: 10.3390/cells10061278 (PMC8224046; doi:10.3390/cells10061278)

Original blots for Fig. 2E.

PKCa/b

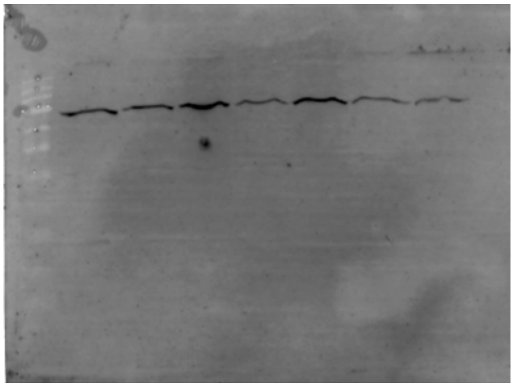

pPKD/PKC m

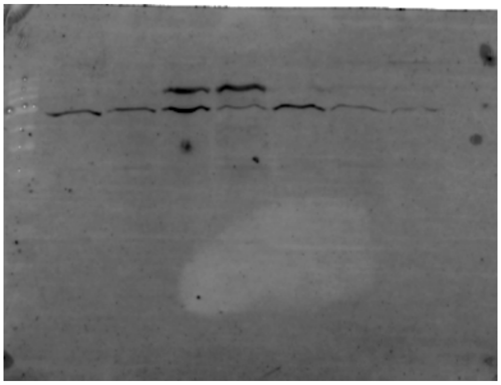

B-actin

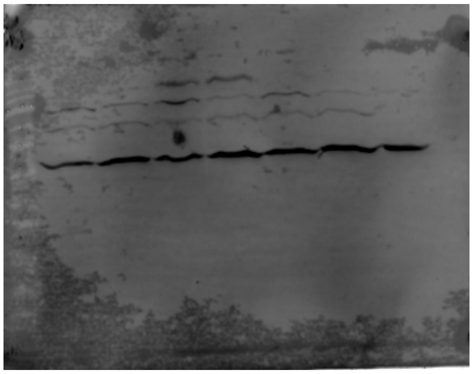

Original blots for Fig. 5A.

Complex I

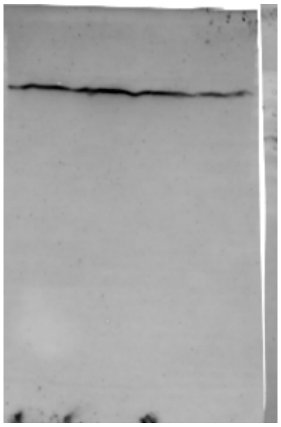

Complex III

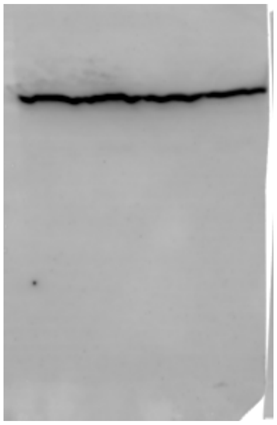

Complex IV

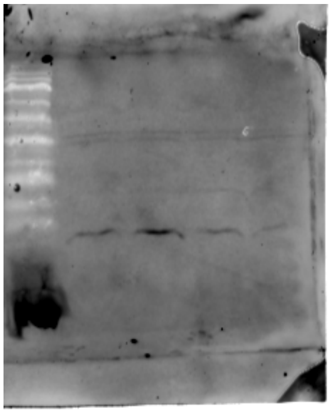

Complex V

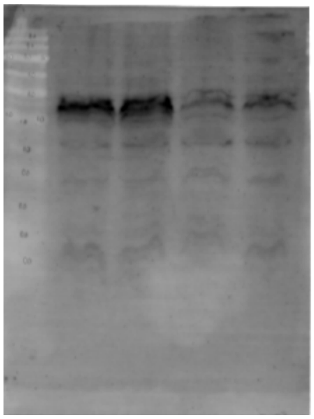

VDAC

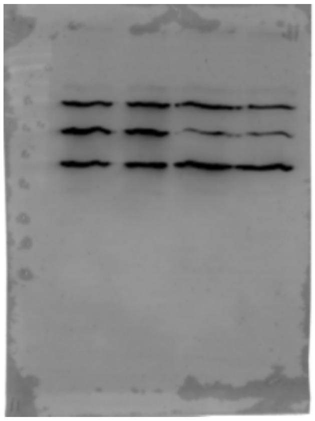

Original blots for Fig. 6D

pAMPK

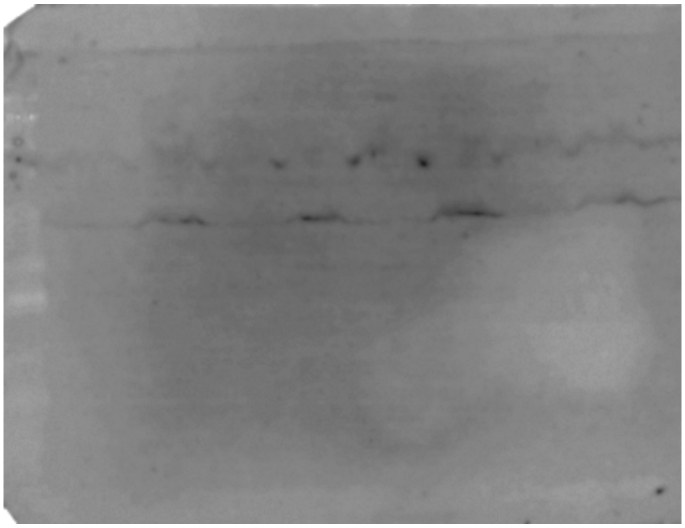

AMPK

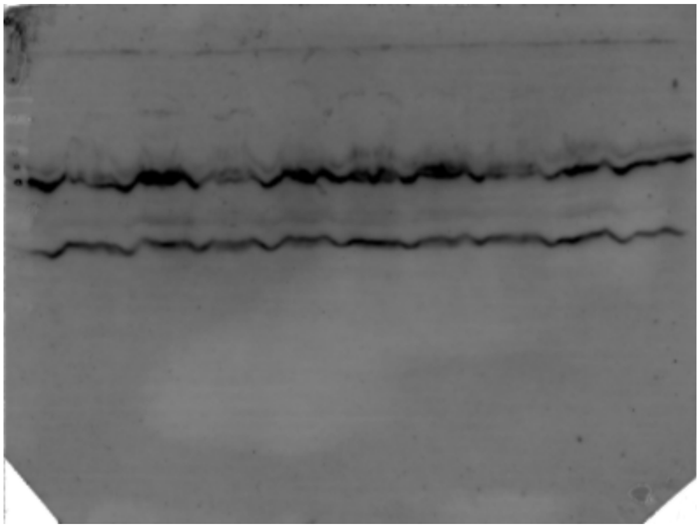

Supplement: Supplementary file 1 [file cells-10-01278-s001.zip › cells-1185038-supplementary.pdf]
